# An Automatic Embedded Device Registration Procedure Based on the OGC SensorThings API

**DOI:** 10.3390/s19030495

**Published:** 2019-01-25

**Authors:** Chih-Yuan Huang, Hsin-Hsien Chen

**Affiliations:** 1 Center for Space and Remote Sensing Research, National Central University, Taoyuan 320, Taiwan; 2 Department of Civil Engineering, National Central University, Taoyuan 320, Taiwan; s83527@g.ncu.edu.tw

**Keywords:** Internet of Things, sensor web, plug and play, interoperability, OGC SensorThings API

## Abstract

Sensor Web and Internet of Things (IoT) (SW-IoT) have been attracting attention from various fields. Both of them deploy networks of embedded devices to monitor physical properties (i.e., sensing capability) or to be controlled (i.e., tasking capability). One of the most important tasks to realize the SW-IoT vision is to establish an open and interoperable architecture, across the device layer, gateway layer, service layer, and application layer. To achieve this objective, many organizations and alliances propose standards for different layers. Among the standards, Open Geospatial Consortium (OGC) SensorThings API is arguably one of the most complete and flexible service standards. However, the SensorThings API only address heterogeneity issues in the service layer. Embedded devices following proprietary protocols need to join closed ecosystems and then link to the SensorThings API ecosystem via customized connectors. To address this issue, one could first follow another device layer and gateway layer open standards and then perform data model mapping with the SensorThings API. However, the data model mapping is not always straightforward as the standards were designed independently. Therefore, this research tries to propose a more direct solution to unify the entire SW-IoT architecture by extending the SensorThings API ecosystem to the gateway layer and the device layer. To be specific, this research proposes SW-IoT Plug and Play (IoT-PNP) to achieve an automatic registration procedure for embedded devices. The IoT-PNP contains three main components: (1) A description file describing device metadata and capabilities, (2) a communication protocol between the gateway layer and the device layer for establishing connections, and (3) an automatic registration procedure for both sensing and tasking capabilities. Overall, we believe the proposed solution could help achieve an open and interoperable SW-IoT end-to-end architecture based on the OGC SensorThings API.

## 1. Introduction

Sensor Web is a widely distributed network connecting different types of sensors [1,2]. With enough power supply, in-situ or remote sensors can continuously collect observations of instruments and environment [3]. By sharing these large number of observations online via interoperable ICT (Information and Communication Technologies), users can integrate the observations to discover phenomena that were not observable in the past [2].

In recent years, the Internet of Things (IoT), a similar idea to the Sensor Web, attracted much attentions from various fields. While there have been many definitions of the IoT, International Telecommunication Union (ITU) introduced a general yet comprehensive definition. The ITU defines that the IoT is “a global infrastructure for the information society, enabling advanced services by interconnecting (physical and virtual) things based on existing and evolving interoperable information and communication technologies”, and a thing is “an object of the physical world (physical things) or the information world (virtual things), which is capable of being identified and integrated into communication networks”.

As the Sensor Web and the Internet of Things are similar in terms of their architectures and functionalities, we name them as the SW-IoT (Sensor Web and IoT) in this paper. In general, the SW-IoT provides two main functionalities [4]:

(1) Sensing capability: SW-IoT devices can monitor their latest status or use embedded sensors to observe properties of their surrounding environment. With the help of communication technologies, users can remotely access these observations for further analysis or decision-making.
(2) Tasking capability: Tasking capabilities allow users to remotely control SW-IoT devices. For example, a client can send tasks to turn on/off a device, adjust the sensor’s sampling rate, schedule an observation event, etc.

The integration of sensing and tasking capabilities helps achieve various applications, such as precision agriculture, Industry 4.0, smart city, home automation, and e-Health, etc. In general, we can summarize the SW-IoT with a three-tier architecture [5], which contains the device layer, the service layer, and the applications layer. In terms of sensing capabilities, devices embedded with sensors can continuously collect and upload observations to services on the service layer. The service layer mainly supports data management and interfacing while the application layer retrieves data from the service layer for further applications.

In terms of tasking capabilities, a client in the application layer can first send tasks to the service layer. A service then transforms the tasks into a device request by following a corresponding device protocol. Once the service forwards the request to the device, the device will execute the task accordingly.

**Figure 1:**
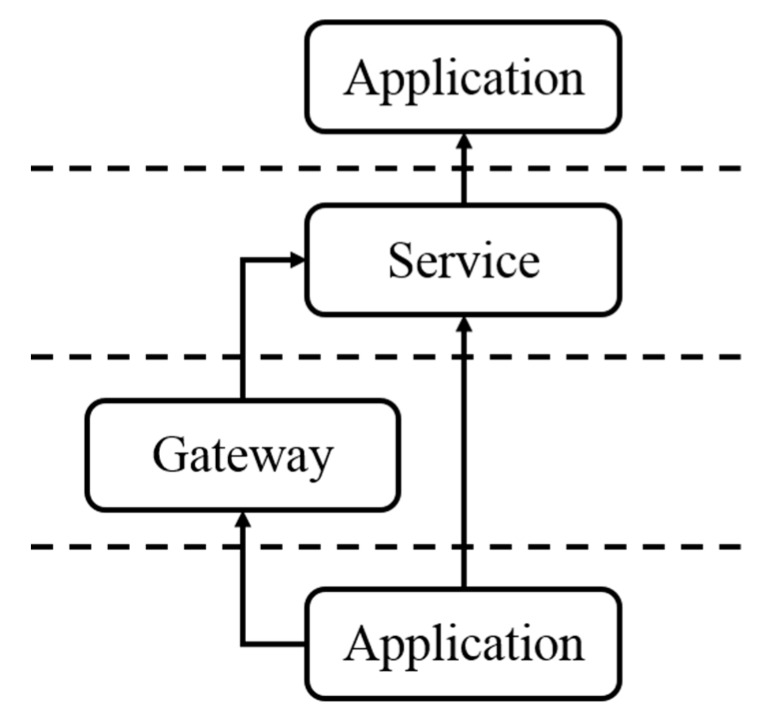
A high-level Sensor Web and Internet of Things (SW-IoT) architecture [4].

Besides the three aforementioned layers, an additional gateway layer is commonly applied in the SW-IoT systems (Figure 1). The gateway layer serves as an intermediate between the device layer and the service layer. There are two advantages to applying gateways. Firstly, while most embedded devices are resource-constraint and not able to connect to the Internet by themselves [6], gateways can help devices to communicate with services. Secondly, as directly connecting to the Internet requires higher energy consumption, devices can connect with gateways via low-power-consumption wireless communication protocols [7], such as Bluetooth LE, ZigBee, LoRaWAN, and NB-IoT, etc. 

With the advance of micro-controller and sensor technologies in recent years, the cost of constructing SW-IoT systems has been significantly reduced. Government agencies, scientists, and developers can customize and deploy embedded systems at a lower cost. However, instead of following open standards, many SW-IoT systems are constructed based on proprietary protocols due to its conveniences. These systems form many SW-IoT silos that cannot communicate with each other directly. This heterogeneity issue has been identified as one of the most critical issues severely obstructing the development of SW-IoT [3]. An ultimate solution for this issue is to unify the communication protocols between different layers by following mutually agreed standards. Among the standards related to the SW-IoT, the Sensor Web Enablement (SWE) standards defined by the OGC (Open Geospatial Consortium) have been widely adopted [8,9,10,11]. The SWE mainly defines web service interface standards, such as SOS (Sensor Observation Service) [12], SPS (Sensor Planning Service) [13], SensorThings API [14], and data model standards, such as O&M (Observations and Measurements) [15], SensorML (Sensor Model Language) [16]. By following the SWE standards, sensor metadata and observations can be described with a unified framework, and sensing and tasking capabilities can be accessed in an interoperable manner.

In addition, among the SWE standards, the SensorThings API (Application Programming Interface) is the newest RESTful service interface standard for SW-IoT data services. This standard has two main advantages. Firstly, this standard defines a comprehensive SW-IoT data model based on the O&M standard. Secondly, its service interface is inspired by the OASIS (Organization for the Advancement of Structured Information Standards) Open Data Protocol (OData) [17], which supports intuitive and flexible query functions based on the Representational State Transfer (RESTful) style and JavaScript Object Notation (JSON) encoding. Therefore, we believe that the SensorThings API is one of the most complete SW-IoT data service interfaces.

However, while the SWE standards mainly focus on the service layer, communication between devices and gateways did not receive enough attention in the SWE ecosystem. To be specific, the communication between the device layer and the gateway layer is important for device registration, observation uploading, and task forwarding. Although there have been some efforts proposed to address this issue in the SWE ecosystem, such as PUCK [18] and SID (Sensor Interface Descriptors) [19], these approaches are either not open standards or are not designed to integrate with the SensorThings API.

Therefore, the main goal of this research is to fill in the missing components in the current SensorThings API architecture, which allows embedded devices to automatically register themselves and their sensing and tasking capabilities to a SensorThings API service through local gateways. This solution is designed to support various scenarios. The scenarios can be summarized into four general types, including the registration of sensing capabilities of in-situ and mobile devices, and the registration of tasking capability of in-situ and mobile devices. To be specific, in-situ and mobile devices can automatically register themselves to a SensorThings API service, continuously upload sensor observations or new locations, and update web addresses of connected-gateways for services to task devices. These scenarios should be able to cover most SW-IoT use cases via the integration of sensing and tasking capabilities [20]. In general, this research tries to construct a coherent architecture that can seamlessly integrate the device layer, the gateway layer, and the service layer based on the SensorThings API ecosystem.

To be specific, we propose a solution called SW-IoT Plug and Play (IoT-PNP) to achieve an automatic registration procedure from embedded devices to SensorThings API services. There are three main parts in the IoT-PNP. Firstly, this research defines a description file format for device metadata and capabilities, in order that the devices become self-describing. Secondly, the IoT-PNP defines a default configuration of a local wireless communication protocol to automatically establish connections between devices and gateways. Thirdly, registration procedures for sensing and tasking capabilities are designed to support different possible scenarios, which are new devices, new gateways, and mobile devices.

In general, the contributions of this research are as follows. First of all, the proposed IoT-PNP can fill in the missing components of the SensorThings API ecosystem to construct a complete SW-IoT architecture covering the device, gateway, and service layers. Second of all, the proposed registration procedures can support various scenarios for sensing and tasking capabilities on in-situ and mobile devices. Third of all, by applying a keyword replacement mechanism, the proposed solution can automatically translate tasking requests to heterogeneous device protocols. However, similar to every approach that aims to achieve interoperable communication, a certain degree of modification on devices are required to support the proposed solution.

In the next section, we review existing solutions proposed by different organizations to address heterogeneity issues on different layers of an SW-IoT system and analyze the advantages and disadvantages of these solutions. In Section 3, we introduce our solutions, the SW-IoT plug-and-play (IoT-PNP). In Section 4, we apply the IoT-PNP on four use-cases based on different scenarios. Finally, Section 5 concludes this research.

## 2. Related Work

To address the aforementioned SW-IoT heterogeneity problem, there are two possible approaches. The first one is the hub approach. The hub approach is achieved by implementing different connectors corresponding to different SW-IoT ecosystems. With the connectors, a hub can communicate with different systems. A hub can be in the application layer, service layer or gateway layer as long as the hub can support protocols of different systems. For example, If-This-Then-That (IFTTT), a service hub on the application layer, supporting many connectors connecting to many web applications and SW-IoT systems. The hub approach is effective and easy to develop. However, for as many SW-IoT systems, a hub needs to implement customized connectors, which results in significant development cost.

The second approach is the standard approach. In principle, by following standards, SW-IoT systems from different manufacturers can collaborate with each other within each standard ecosystem. There are some organizations proposing their proprietary standards, such as Apple’s “Works with HomeKit (https://www.apple.com/tw/ios/home/accessories/)” and Google’s “Works with Nest (https://nest.com/works-with-nest/)”. Different from the hub approach, enterprises are trying to define proprietary standards to attract manufacturers to join their ecosystems. However, proprietary standards and ecosystems would cause more heterogeneity issues as no existing SW-IoT ecosystem is large enough to attract all manufactures. On the other hand, the real potential of the standard approach is to follow open and commonly agreed standards.

For example, there are many existing open standards defined for unifying interfaces of the SW-IoT Service Layer. Among the standards, the OGC SOS is a web service interface standard for sensor resource sharing. Information shared by SOS services is described with other SWE standards such as the O&M and SensorML. However, the SOS is based on Simple Object Access Protocol (SOAP) and XML-based network protocol, which are considered as outdated and inefficient. Therefore, OGC recently defines another service interface standard, the SensorThings API. The first part of the SensorThings API standard is primarily designed for the SW-IoT sensing capability. Different from other OGC service interfaces, the SensorThings API applies RESTful style and JSON encoding to provide a more lightweight and efficient interface. The data model of the SensorThings API is mainly extended from the O&M data model by including classes of IoT to ensure the versatility of its data model. The interface of the SensorThings API service is inspired by the OASIS OData. SW-IoT resources are represented as entities or properties that can be identified by URIs (Uniform Resource Identifiers), and resources can be linked together using relationship links. The second part of the SensorThings API is under development and focuses on extending the data model and service interface to support tasking capabilities.

In addition, there are some standards designed by different industrial alliances. HyperCat standard [21] is proposed by the HyperCat consortium, an organization which is composed of more than 40 UK-based companies including British Telecom, Rolls-Royce, and military supplies manufacturer BAE, etc. The HyperCat is an open, lightweight, JSON-based online catalog tagged with metadata. The HyperCat exposes SW-IoT devices’ information and observation resources over the web with semantic annotations. Instead of requiring manufacturers to follow the same data format, the HyperCat can describe data from different providers and help users search for the data relevant to their needs.

OpenIoT standard [22] is a European Union (EU) open source web service standard released by developers from industry and academia jointly in 2012, which uses semantic web technologies to connect physical and virtual sensors to the OpenIoT web platform. The OpenIoT utilizes the Extended Global Sensor Network (XGSN) [23] as a hub between the OpenIoT service and the physical world, which can collect and filter semantically annotated data streams from SW-IoT devices.

On the other hands, some open standards focus on device layer protocols. OneM2M is an international standard organization composed of standard organizations from Europe, America, China, Japan, and South Korea, and was established in July 2012. The oneM2M standard designs a distributed software layer that provides an interoperable framework. This distributed software layer is composed of three entities, Application Entity (AE), Common Services Entity (CSE), and Network Services Entity (NSE) [24]. An AE is an application implemented on oneM2M nodes. A CSE provides a set of common services functions (CSFs) to other AEs, CSEs, NSEs. An NSE provides network service functions from underlying networks such as 3GPP, 3GPP2, and WLAN to the CSEs.

Furthermore, OGC PUCK is a standard that aims at providing interoperable communications between devices and host computers through serial cables (RS-232) or Ethernet. PUCK defines several PUCK commands for hosts to discover PUCK-enabled devices and retrieve devices’ metadata or drivers from devices. The hosts can parse devices’ metadata by following the PUCK standard format and install drivers to communicate with devices. Another solution based on the OGC SWE ecosystem is Sensor Interface Descriptor (SID). The SID aims at solving the heterogeneity issue between the service layer and the device layer [19]. The SID defines a schema and an extension to the OGC SensorML, to describe a sensor’s communication protocol, commands, processing steps, and metadata association. According to the SID, a SID interpreter (gateway) can translate a sensor protocol into the OGC web services interface. A similar work to the SID was proposed by Martínez et al. [25], where the authors defined the Sensor Deployment Files (SDF) describing the metadata of a sensor and designed a framework to retrieve SDF via PUCK and register information to OGC SOS.

Universal Plug and Play (UPnP) [26] is a network protocol proposed by the UPnP forum, which aims at providing seamless connections for devices in the same Local Area Network (LAN). The UPnP is a distributed architecture based on the TCP/IP, HTTP, XML, and SOAP network. The UPnP provides “Addressing” and “Discovery” procedure for new devices and services to join the UPnP network. A control point can control devices or subscribe to events of devices according to the description provided by devices.

As mentioned earlier, the objective of this study is to achieve automatic connections between the service layer and the device layer. The OGC SOS, SensorThings API, OpenIoT are standard service interfaces for the service layer. The HyperCat provides a catalog service and data semantic annotation for sensor data searching, where the annotation and publish should be manually finished. The OGC PUCK mainly defines commands for device communication, where an automatic registration procedure was not defined. Some manual work is still needed for sharing sensing and tasking capabilities to the service layer. The SID provides a complete data model for describing sensing and tasking capabilities. However, for the Plug and Play, their data model is verbose in XML schema and is complicated for devices to support. The SDF approach is also based on XML and only supports sensing capabilities. The UPnP operates on networks based on the TCP/IP, HTTP, XML, and SOAP, thus that the standard cannot be applied to devices on low-power wireless communication networks. The oneM2M provides an operating-system-like logic layer for various kind of devices. However, as the oneM2M only defines a general and flexible data model, integrating sensor information from different devices may have serious heterogeneity issues.

Based on our understanding, the existing solutions cannot fully address the heterogeneity issue between the Service Layer and the Device Layer. Therefore, this research aims at proposing a solution to allow embedded devices to be self-describing, automatically establish a connection between devices and gateways via local wireless communication protocols, register device’s sensing and tasking capabilities to the service layer, and control devices via the service layer and the gateway layer. In addition, the OGC SensorThings API is chosen as the service layer because of its comprehensive data model and flexible service interfaces.

## 3. Methodology

This research proposes the SW-IoT plug-and-play (IoT-PNP) to automatically establish communication between the device layer and the service layer for both sensing and tasking capabilities of embedded devices. The IoT-PNP has three main components: (1) A data model and encoding describing devices’ metadata, sensing and tasking capabilities, (2) a communication protocol and default connection settings for the gateway layer and the device layer, and (3) an automatic registration procedure for registering devices and their sensing and tasking capabilities on a SensorThings API service. The details of these components are explained in the following subsections.

### 3.1. Device Description Data Model and Encoding

#### 3.1.1. OGC SensorThings API Data Model

**Figure 2:**
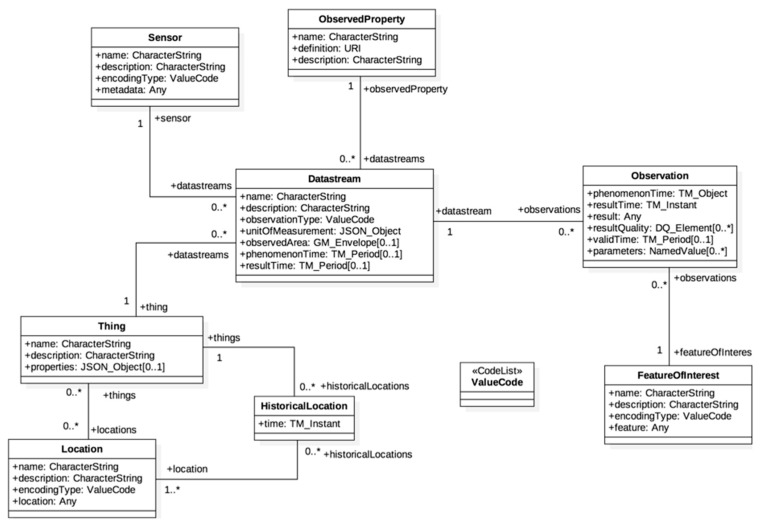
The SensorThings API UML diagram [14].

In the OGC SensorThings API, devices can be described by a standard data model, which is extended from the OGC O&M model. The O&M defines a complete data model of an observation event by identifying necessary classes/properties and their relationships. The SensorThings API includes the SW-IoT concept into the O&M data model by introducing additional classes. Figure 2 is the UML (Unified Modeling Language) diagram of the SensorThings API data model. An SW-IoT device can be modeled as a Thing entity. A Thing uses Location entities to introduce its current location and uses HistoricalLocation entities for its past trajectory. A Thing can have one or more Datastreams, where each Datastream is a logical aggregation of sensor Observations of an ObservedProperty produced by the same Sensor. Observations may observe different FeaturesOfInterest. As the O&M data model has been applied in many fields for more than a decade, this SensorThings API data model is also considered complete and general for the SW-IoT.

#### 3.1.2. Extended SensorThings API

**Figure 3:**
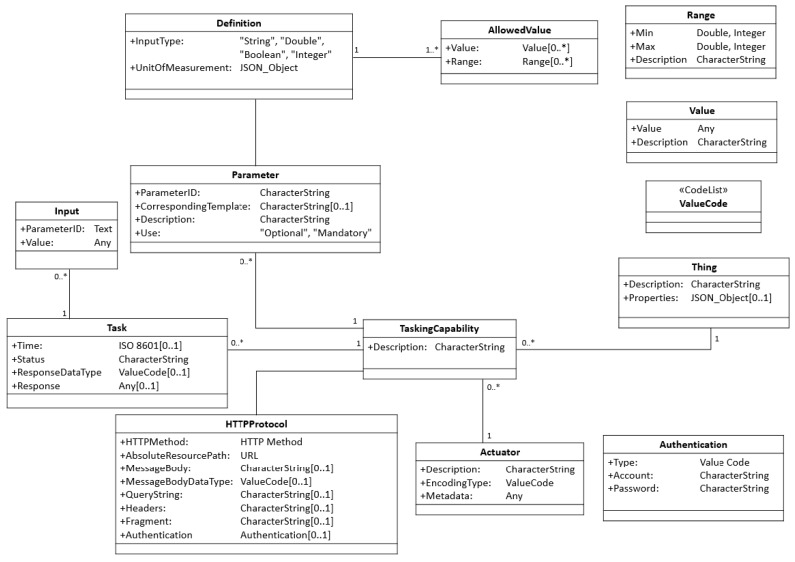
The data model of tasking capability [4].

As mentioned earlier, the first part of the SensorThings API is only designed for the sensing capability but did not consider the tasking capability. To address this issue, Huang and Wu [4] proposed the Extended SensorThings API (Figure 3) as a potential solution to support tasking capabilities in the SensorThings API ecosystem. Firstly, the data model is extended, where each Thing can have zero to many TaskingCapability entities. Each TaskingCapability is executed by an Actuator. When a client can create Tasks based on applicable Parameters of a TaskingCapability, an Extended SensorThings API service will apply the HTTPProtocol of the TaskingCapability to translate Tasks into requests following the device protocol. To be specific, Huang and Wu [4] proposed the keyword replacement solution to perform automatic translation between the device protocol and the SensorThings API protocol. Based on this solution, no human intervention is required as long as devices can describe their tasking capabilities based on the defined data model.

Comparing with the original SensorThings API standard, the Extended SensorThings API provides an extended data model to describe tasking capabilities of SW-IoT devices. Based on the data model, users can remotely control devices through Extended SensorThings API services. As OGC is still defining the second part of the SensorThings API, this research applies the Extended SensorThings API [4] as the Service Layer to support both sensing and tasking capabilities.

#### 3.1.3. Description File

In terms of the proposed IoT-PNP, to automatically register embedded devices to the service layer, embedded devices need to be self-describing, which means metadata, sensing and tasking capabilities, and device communication protocols should be introduced by the devices themselves. Therefore, the first component of the IoT-PNP is to define a description file to explain the details of embedded devices. Since this research aims at proposing a plug-and-play solution based on the SensorThings API, we can directly follow the SensorThings API data model to design the description file. The following subsections explain three sections of the description file, which are Thing’s metadata, sensing capabilities, and tasking capabilities.

##### Description File—Thing Metadata

**Figure 4:**
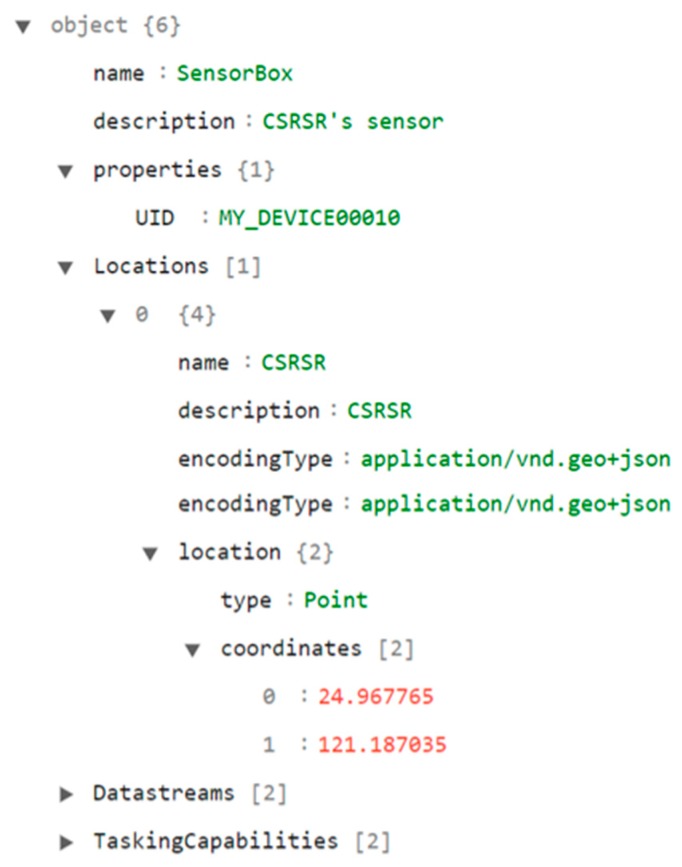
Thing metadata of an example description file.

This section introduces the metadata of an embedded device, such as name, properties, and locations. Figure 4 shows an example of the Thing metadata section. According to the SensorThings API definition, the properties of a Thing are key-value pairs for developers to describe any information related to this Thing. For this research, to support the automatic registration procedure, we define a new property called UID that indicates a unique identifier of a device. This identifier should be unique at least among all devices in a single service. In the automatic registration procedure, the UID is applied to identify the registration status of a device. Please note that the name property of a Thing entity could also serve the same purpose if the name property is defined as unique in a service.

##### Description File—Sensing Capability

**Figure 5:**
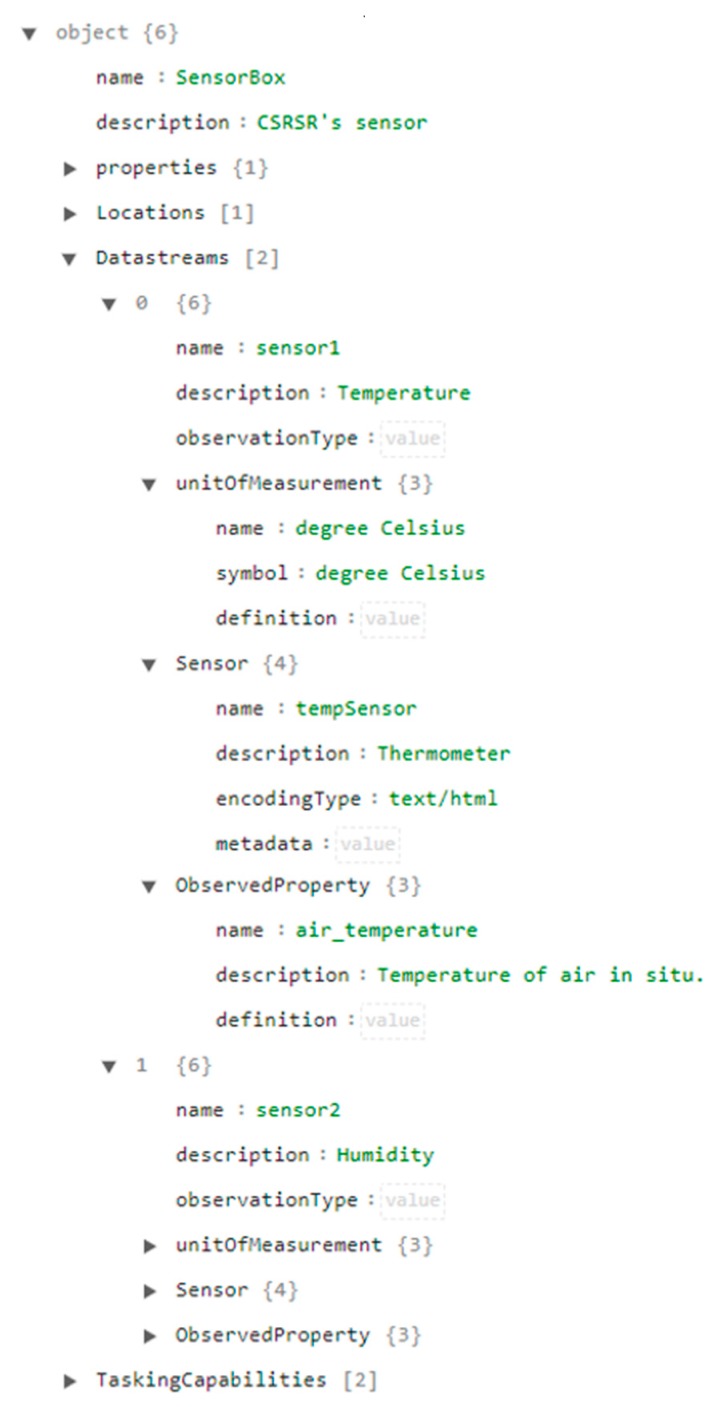
Sensing capability of an example description file.

By following the SensorThings API data model, sensing capabilities can be directly modeled as Datastream entities. According to the SensorThings API definition, one Datastream links to a Sensor and an ObservedProperty. Therefore, the information of the corresponding Sensor and ObservedProperty should also be described in the description file along with other necessary attributes, such as the unitOfMeasurement. After registering a Datastream to a service, Observations of this Datastream will be created and linked to this Datastream entity. Figure 5 shows an example description file where one device has two Datastreams measuring air temperature and humidity.

##### Description File—Tasking Capability

**Figure 6:**
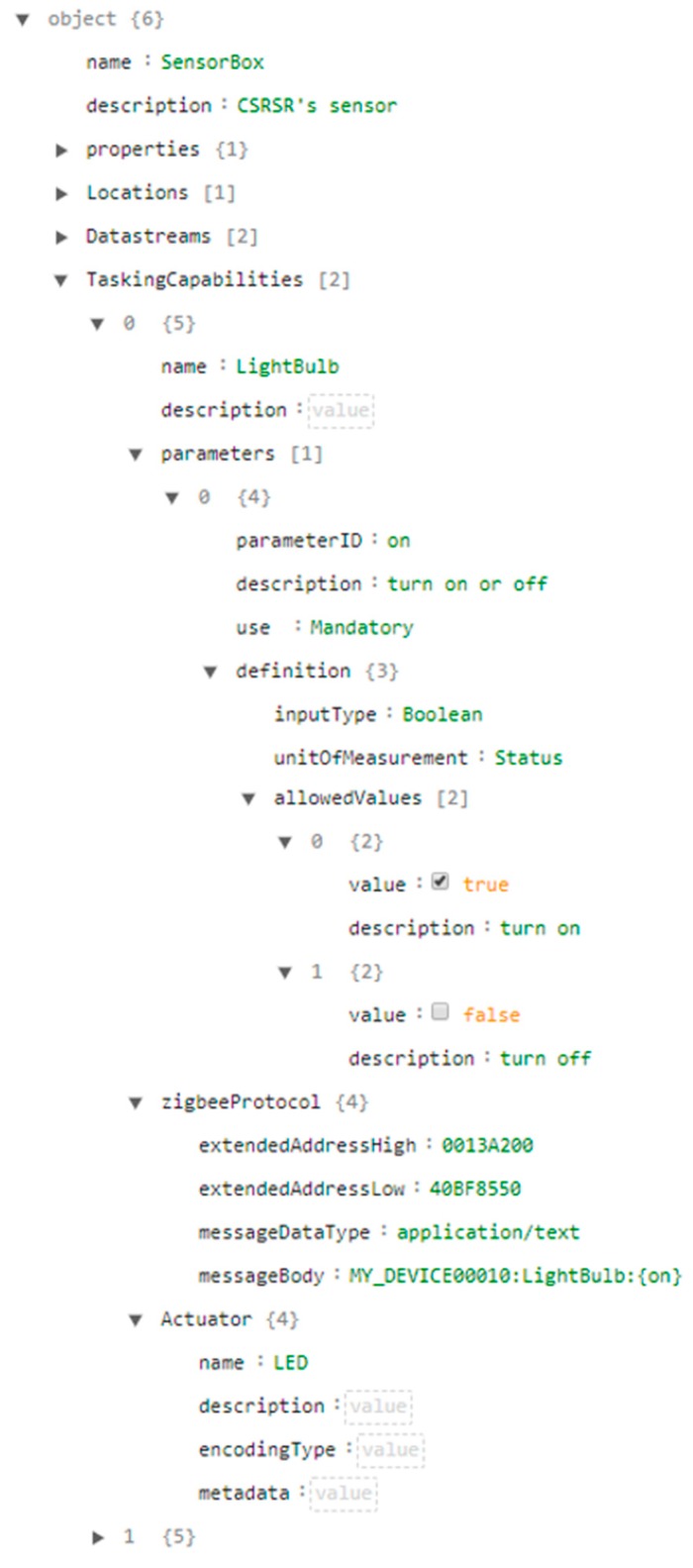
Tasking capability of an example description file.

As mentioned earlier, this research applies the data model defined in the Extended SensorThings API (Figure 3) to support the tasking capability. However, since the Extended SensorThings API was designed as a service interface, this description file is mainly for embedded devices to introduce themselves to gateways. Therefore, instead of specifying the HTTPProtocol, this description file should explain the device protocol on local wireless communication protocols, such as ZigBee, Bluetooth, or Low Power WANs. To be specific, the HTTPProtocol should be replaced by classes like ZigBeeProtocol or BluetoothProtocol, etc. As this research chooses ZigBee as the local wireless communication protocol for implementation, the description file with the proposed ZigBeeProtocol is shown in Figure 6.

In the ZigBeeProtocol, extendedAddressHigh and extendedAddressLow are the high and low 32 bits of unique IEEE 64-bit Extended Address of a device’s ZigBee module to establish a connection between gateways and devices. The messageBody is a device’s tasking request template, as messageDataType describes the data type of messageBody. The tasking capability in this research also adopts the keyword replacement strategy proposed by Huang and Wu [4] to describe the device protocol and acceptable parameters. The content of messageBody can be defined by device developers as long as it follows the rule of keyword replacement, which will be explained in detail in Section 3.2.3. Finally, similar to the sensing capability, as one device can support different tasking capabilities, a Thing could have multiple TaskingCapability entities. Figure 6 shows an example description file where one device has a TaskingCapability serving as a smart light bulb that can be turned on/off via ZigBee.

### 3.2. Communication Protocol for the Gateway Layer and the Device Layer

As the middle layer between the service layer and the device layer, the gateway layer should provide three functionalities: (1) Allowing devices connected to a gateway through different wireless communication protocols, (2) interpreting a device’s description file and registering the device and its observations to a specified SensorThings API service, and (3) translating the extended SensorThings API tasking protocol to the device tasking protocol. To achieve these functionalities, this research defines: (1) Default configurations of a wireless communication protocol, which is ZigBee in this research, (2) communication operations on gateways and devices, and (3) an automatic procedure for tasking protocol translation and task forwarding. These three designs are introduced respectively in the following subsections.

#### 3.2.1. ZigBee Configuration

To wirelessly connect to the Internet, Wi-Fi was designed for wireless Ethernet connectivity. However, Wi-Fi modules have high power consumption for embedded devices to support. Therefore, wireless network techniques with low power consumption are more preferable for embedded devices, such as Bluetooth, ZigBee, LoRa, SigFox, NB-IoT, etc. Therefore, in the SW-IoT architecture, a gateway should communicate with devices via these low-power-consumption wireless communication protocols and connect with services via Wi-Fi or Ethernet. In this research, we choose ZigBee as the wireless communication network to prove the concept.

ZigBee [27] is based on the IEEE 802.15.4 standard [28]. The IEEE 802.15.4 standard focuses on low energy consumption, low-rate transmission, and low-cost wireless network to provide low-speed interconnection between different devices. As the transmission distance of ZigBee is about 10~100 meters, ZigBee is suitable for many small-scale SW-IoT use cases, such as smart home, e-health, industrial 4.0.

According to the IEEE 802.15.4 standard, a ZigBee network has full-function devices (FFDs) and reduced-function devices (RFDs). An FFD can communicate with both FFDs and RFDs. An RFD can only communicate with FFDs. An FFD associated with an RFD is called the coordinator of that RFD. There is an only FFD, called PAN (Personal Area Network) coordinator, in one ZigBee network, which is the main controller of that network. The PAN coordinator is responsible for managing other FFDs (i.e., routers) and RFDs (i.e., end devices) in the network. The PAN coordinator needs to select a PAN ID (16-bit and 64-bit) and a channel to establish the network. All devices in one ZigBee network share the same PAN ID and channel with the PAN coordinator. While the 16-bit PAN ID is randomly selected when a PAN coordinator starts a new network, the ZigBee Alliance defines the 64-bit PAN ID (i.e., Extended PAN ID) which can be preconfigured on a PAN coordinator. Other devices that are configured with the same 64-bit PAN ID can join the same network.

Each ZigBee device possesses 16-bit and 64-bit addresses. The 16-bit address, also called a network address, is randomly generated by the coordinator that allows the device to join the network. However, the 16-bit address is not static, which is sometimes not reliable for data transmission. On the other hand, the 64-bit address, also called extended address or IEEE address, is a universally unique identifier for a ZigBee device, which is a better choice to identify destination devices.

Table 1 shows our design for the IoT-PNP default ZigBee network configuration. First of all, to allow devices to connect to gateways automatically, the wireless communication modules of both devices and gateways should share the same PAN ID. Gateways and devices which want to support the IoT-PNP should follow the defined extended PAN ID (i.e., 14,647). Second of all, based on the ZigBee standard, to configure ZigBee network that (1) the PAN coordinator forwards messages to all devices (i.e., broadcast mode) and (2) messages from devices are transmitted/forwarded to the PAN coordinator, where we define that the PAN coordinator uses “0xFFFF” as the extended address of destination to broadcast messages to the entire network, and routers and end devices use “0” as the extended address of destination to forward messages to the PAN coordinator.

Please note that the role of this configuration is intended to be a global or national setting to establish a public gateway infrastructure, and the current ZigBee configuration is defined to prove the concept. However, this public configuration would result in security concerns such as intercepting messages with malicious gateways. Mechanisms like payload encryption, gateway verification, random/dynamic configurations of local communication protocols for private connections, etc. should be further investigated, which is also a main future work of this research.

#### 3.2.2. Communication Operations on Gateways and Devices

By following the IoT-PNP default ZigBee configuration, devices can automatically join the ZigBee network of IoT-PNP and connect to IoT-PNP gateways. For devices and gateways to exchange information, operations on gateways and devices need to be defined. Each operation is composed of several properties as shown in Table 2. The details of the designed gateway and device operations are explained in the following subsections. The use of these operations is explained in Section 3.3.

##### Gateway Operations

The gateway operations define interfaces allowing devices to send requests to gateways. In general, we have designed five gateway operations, which are UploadObs, UpdateStatus, SendServURL, SendDesc, and SendServURLandDesc. The details of these operations are explained in Table 3, Table 4, Table 5, Table 6 and Table 7. In addition, we use the gray background color to indicate the placeholders to be replaced with actual content, such as the “<device_ID>”.

##### Device Operations

Device operations define interfaces allowing gateways to send requests to devices. We have designed four device operations, which are GetServURL, GetDesc, GetServURLandDesc, and Confirm. The details of these operations are explained in Table 8, Table 9, Table 10 and Table 11. We also use the gray background color to indicate the placeholders to be replaced with actual content.

##### 3.2.3. Tasking Protocol Translation and Task Forwarding

According to the extended SensorThings API, Huang and Wu [4] propose the keyword replacement solution to automatically translate users’ tasks into different device protocols. While Huang and Wu [4] define the interface translation between the service interface and the device protocol, this research applies the same concept to establish automatic protocol translation between the web service and gateway as well as gateway and device.

In general, tasking protocol translation can be divided into the registration process and the tasking process. The registration process has the following steps: (1) A device sends a TaskingCapability entity in the description file to a gateway, (2) the gateway follows the content in local wireless communication protocols, e.g., zigbeeProtocol, and generates a corresponding httpProtocol, and (3) the gateway registers the httpProtocol to an extended SensorThings API service. The tasking process has the following steps: (1) A user creates a Task in the extended SensorThings API service based on the TaskingCapability shown in the service, (2) the service follows the httpProtocol to translate into a gateway request and forward the task to the gateway, (3) the gateway parses the tasking parameters from the service request, (4) the gateway follows the zigbeeProtocol to translate into a device request and forward the task to the device.

We use a smart light bulb example in Figure 6 to explain the detail procedure. First of all, the device sends the describe file in Figure 6 to a gateway. Based on the TaskingCapability entity, the gateway can understand there is a task template, i.e., MY_DEVICE00010:LightBulb:{on}, with a placeholder {on}. The on in the placeholder {on} is the parameterID introduced in the TaskingCapability entity of this light bulb, which will be replaced as true or false to turn on or off the light bulb. By following the content in the TaskingCapability entity, the gateway then replaces the zigbeeProtocol to a httpProtocol as the gateway protocol. The absoluteResourcePath is the static IP address of the gateway. Please note that the httpProtocol can be defined by gateway developers as long as the gateway can understand the mapping between the zigbeeProtocol and the httpProtocol. Algorithm 1 shows an example of an httpProtocol created by the gateway, where the gateway still uses {on} as the placeholder in the httpProtocol.

**Algorithm 1.** An httpProtocol example.

"httpProtocol": {
"httpMethod": "POST",
  "absoluteResourcePath": "http://example.com/Gateway",
  "messageDataType": "application/json",
  "messageBody":
  "{\"on\":**{on}**,\"TC_name\":\"LED\",\"device_ID\":\"DEVICE00001\"}"
}


When a client sends a task (Algorithm 2) to an extended SensorThings API service, the service replaces the placeholder {on} with an input value true specified in the client’s request, and sends an HTTP request (Algorithm 3) to the gateway. After the gateway receives the HTTP request, the gateway finds out that the placeholder {on} has been replaced with true, and then follows the messageBody in the zigbeeProtocol to replace the placeholder {on} with true as well. Finally, the gateway sends the translated task (Algorithm 4) to the device by following the ZigBee connection settings in the zigbeeProtocol.

**Algorithm 2.** A Task from a user to the service.

{
  "TaskingCapability": {
     "@iot.id": 1
  },
  "input": [
     {
       "ParameterID": "on",
       "Value": true
     }
  ]
}


**Algorithm 3.** The task forwarded to the gateway.

POST http://example.com/Gateway HTTP/1.1
 
{
  "on": **true**,
  "TC_name": "LED",
  "device_ID":
  "DEVICE00001"
}


**Algorithm 4.** The task forwarded to the device.

MY_DEVICE00010:LightBulb:**true**


### 3.3. Automatic Registration Procedure

The communication procedure between the device layer, the gateway layer, and the service layer should be standardized to achieve automatic registration. In the following subsections, we first introduce the possible scenarios and then explain the proposed procedures for sensing and tasking capabilities.

#### 3.3.1. Scenarios

We have considered three possible scenarios that the IoT-PNP procedure would encounter. The first scenario is that a new device wants to join an SW-IoT system via a nearby gateway. The second scenario is that an old gateway is replaced by a new gateway, which needs to collect necessary information from nearby devices in the local network. The third scenario targets on mobile devices, where devices can connect to new gateways when moving to other networks. These three scenarios are also the use cases we applied to examine the proposed solution.

#### 3.3.2. Registration Procedure for the Sensing Capability

**Figure 7:**
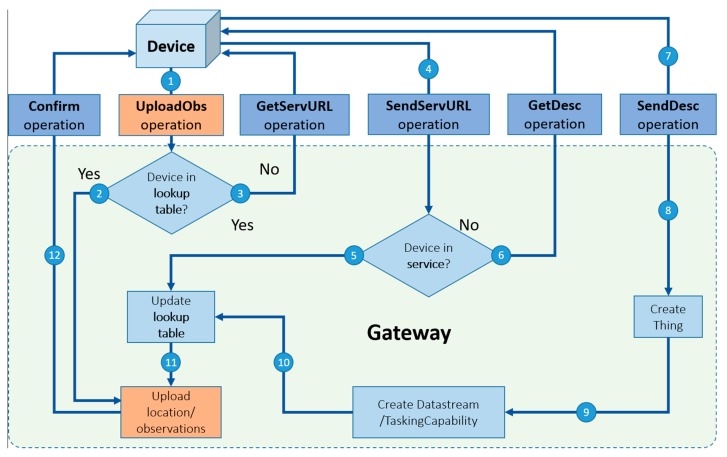
The workflow of the sensing capability registration procedure.

When a device joins an SW-IoT system, the device will be registered in the gateway’s lookup table and the SensorThings API services. The gateway holds a lookup table which records the description files of devices exist in the local network as well as their corresponding virtual identities on the SensorThings API service. Figure 7 is the overall registration procedure for the sensing capability.

1. First of all, the registration procedure is triggered when a device tries to upload observations or new locations by sending an UploadObs operation (Arrow 1) to the connected gateway. The UploadObs operation contains the observations or locations in the msg_body (Table 3).
2. Once the gateway receives the UploadObs operation, the gateway will check if the device has been registered in its lookup table according to the device UID.
3. If the device has been registered in the lookup table, the gateway can directly upload the observations or new locations to the service (Arrow 2) as the device’s virtual identities have also been also stored in the lookup table, and then the gateway sends a Confirm operation (Arrow 12) to the device to confirm the end of the procedure.
4. However, if the device has not been registered in the lookup table (i.e., a new device, a new gateway, or a mobile device), the gateway will send the GetServURL operation (Arrow 3) to the device to ask for the service URL that the device wants to be registered to.
5. The device responds with the SendServURL operation (Arrow 4) containing the service URL. With the service URL, the gateway can query the service based on the device UID, to check whether the device has been registered in the service or not.
6. If the device has been registered in the service (i.e., a new gateway or a mobile device), the gateway can collect the device’s sensing capabilities and virtual identities from the service to update its lookup table (Arrow 5), and finally upload the observations or locations to the service (Arrow 11).
7. If the device has not been registered to the service (i.e., a new device), the gateway will send a GetDesc operation (Arrow 6) to the device to ask for the description file of the device.
8. The device responds with the SendDesc operation (Arrow 7) containing the description file.
9. Based on the description file, the gateway helps the device register to the service and create necessary entities, including Thing (Arrow 8), Datastream, or TaskingCapability (Arrow 9), etc. In this step, the gateway checks if there is already the same ObservedProperties registered on the service. If the ObservedProperties have been registered, the gateway will retrieve the IDs and link new Datastreams to the existing ObservedProperties.
10. Finally, after the gateway uploads the observations or locations to the service, the gateway sends a Confirm operation (Arrow 12) to the device to confirm the end of the procedure.

Please note that although this workflow is mainly for the sensing capability, the gateway registers both sensing and tasking capabilities to the service.

#### 3.3.3. Registration Procedure for the Tasking Capability

**Figure 8:**
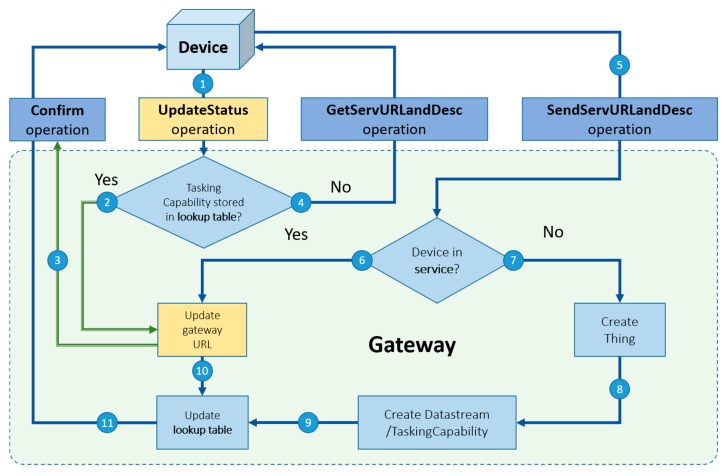
The workflow of the tasking capability registration procedure.

The proposed tasking capability procedure requires gateways to have static IP addresses, which is more realistic than requiring each device to have a static IP address. In our design, gateways receive tasks from services and deliver the tasks to corresponding devices through local communication protocols. Therefore, when a gateway registers a device to a service, the IP address of the gateway and the httpProtocol should be uploaded to the services. However, considering the mobile device scenario, a device could connect to different gateways, which means that the currently-connected gateway should dynamically update the IP address and the httpProtocol on the service. Figure 8 shows the proposed tasking capability registration procedure.

1. First of all, if a device supports any tasking capability, the device should periodically send an UpdateStatus operation (Arrow 1) to the currently-connected gateway.
2. The gateway will first check its lookup table. If the tasking capability of the device has been recorded in the lookup table, which means that this device has been registered to this gateway before, the gateway will directly update the gateway’s IP address and its httpProtocol on the service (Arrow 2) and send a Confirm operation (Arrow 3) to the device to confirm the end of the procedure. The reason of updating the gateway IP address and its httpProtocol even if the device is known to the gateway is that if the device has connected with other gateways (e.g., a mobile device or nearby gateways), the gateway IP address and httpProtocol on the service could have been changed. Therefore, this seen-to-be redundant procedure is to ensure that the service can successfully forward tasks to the gateway.
3. If the gateway cannot find the tasking capability of the device in the lookup table, all three scenarios are possible (i.e., a new device, a new gateway, or a mobile device). However, unlike that gateways can collect devices’ sensing capabilities by querying the service, device protocols (e.g., the zigbeeProtocol) are not stored in the service because of security concerns. Therefore, the gateway needs to send a GetServURLandDesc operation (Arrow 4) to the device to ask for both the service URL and the description file. The device then responds with the SendServURLandDesc operation (Arrow 5).
4. Afterward, the gateway checks if the device has been registered to the service. If the device has been registered (i.e., a new gateway or a mobile device), the gateway will update the gateway’s IP address and its httpProtocol to the service (Arrow 6) and record the device’s sensing and tasking capability in its lookup table (Arrow 10), and finally send a Confirm operation (Arrow 11) to the device to confirm the end of the procedure.
5. If the device has not been registered to the service (i.e., a new device), the gateway will help the device register its sensing and tasking capability to the service (Arrow 7, 8) and its lookup table (Arrow 9), and finally send a Confirm operation (Arrow 11) to the device to confirm the end of the procedure.

Finally, to be specific, all static information required to support the proposed registration procedures could be pre-configured in factories, such as the URL of a SensorThings API service and the description file. However, if manufacturers or applications choose to grant users the ability to modify any information, some application-driven design should be made for users to access and modify the device content via local wireless communication, the SensorThings API service, or other potential means.

## 4. Results

**Figure 9:**
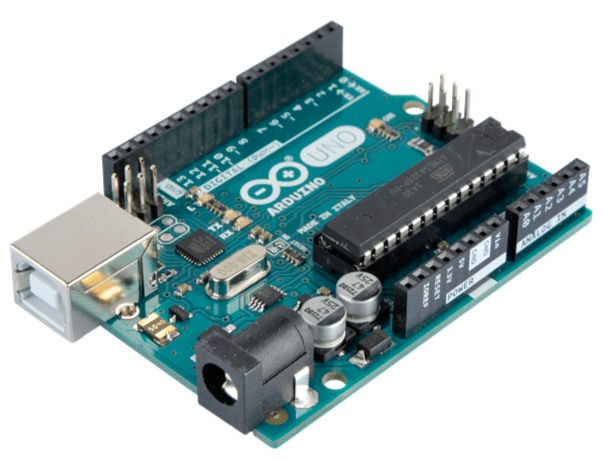
Arduino Uno.

**Figure 10:**
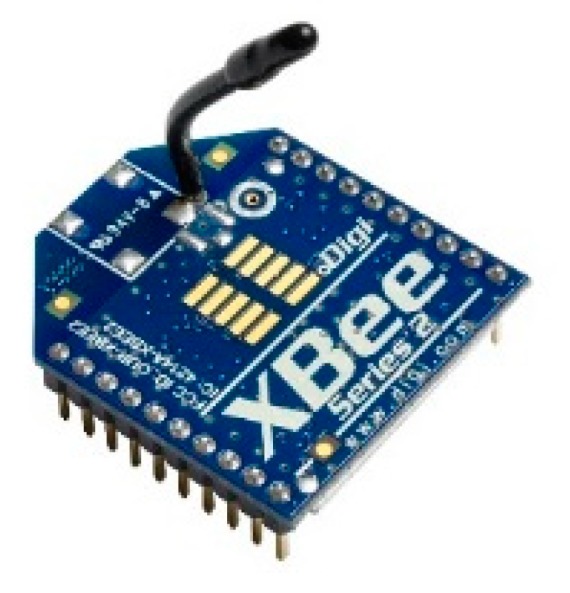
Digi XBee serial 2.

To examine the proposed automatic registration procedure, we design several applications based on different scenarios. As the SW-IoT provides sensing and tasking capabilities and devices can be categorized into in situ or mobile devices, we design four applications: In situ sensing (Section 4.1), in situ tasking (Section 4.2), mobile sensing (Section 4.3), and mobile tasking (Section 4.4). We applied Arduino Uno (Figure 9) as the micro-controller and the Digi XBee serial 2 (Figure 10) for the ZigBee communication. In addition, to evaluate the performance of the proposed solution in a real-world scenario, we have conducted a one-month indoor environment monitoring experiment in an underground metro mall. The details are explained in Section 4.5.

### 4.1. In situ Sensing Application

**Figure 11:**
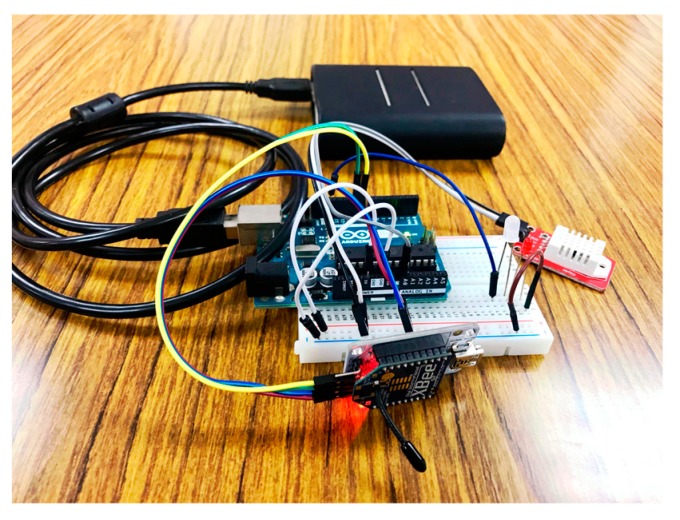
The indoor monitoring device.

**Figure 12:**
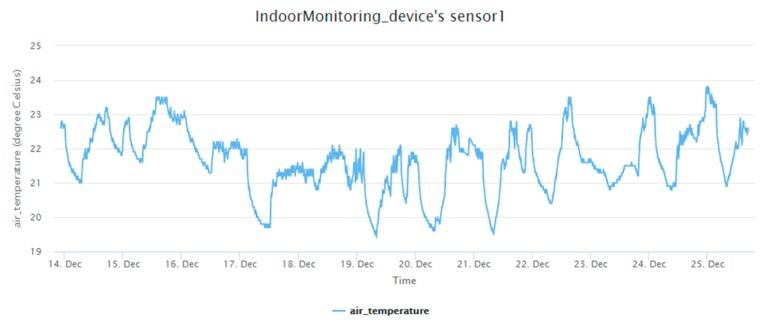
The temperature observations from the indoor monitoring device.

**Figure 13:**
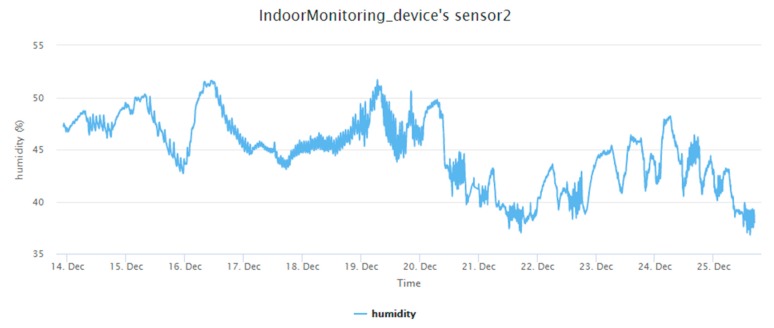
The humidity observations from the indoor monitoring device.

The in situ sensing application we designed is the indoor monitoring. A monitoring device (Figure 11) is installed with a DHT22 sensor measuring temperature and humidity. A gateway, which is a laptop connected with an XBee module, can automatically help new indoor monitoring devices register to the SensorThings API service. In the meantime, the device will periodically measure air temperature and humidity observations and upload the observations via UploadObs requests. Algorithm A1 shows the description file of this embedded device. And Figure 12 and Figure 13 show the time-series observations retrieved from the SensorThings API service.

### 4.2. In situ Tasking Application

**Figure 14:**
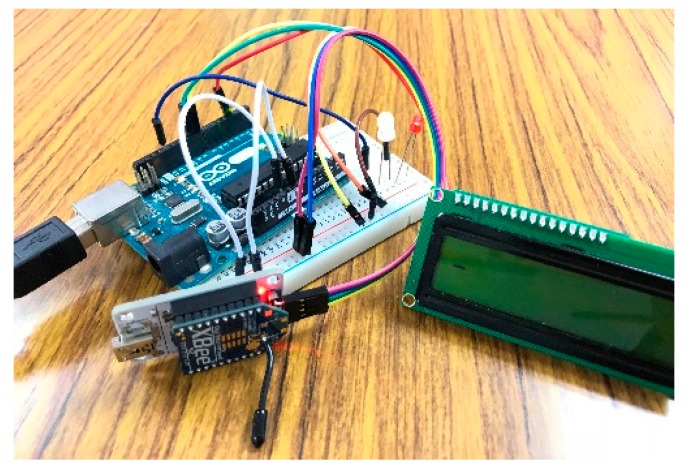
The remotely-controllable Liquid-crystal Display (LCD) and Light-emitting Diode (LED) device.

**Figure 15:**
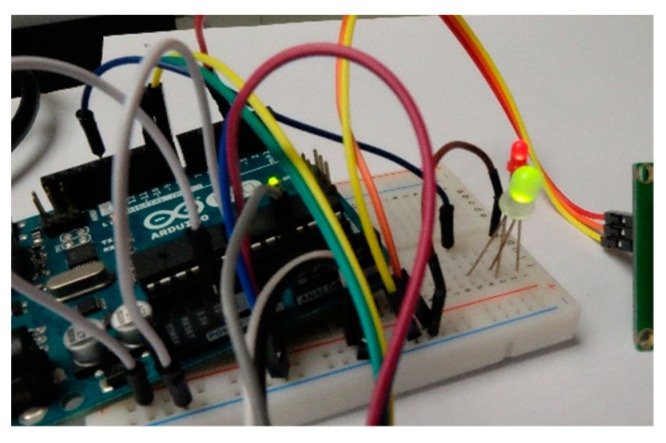
LED turned on.

**Figure 16:**
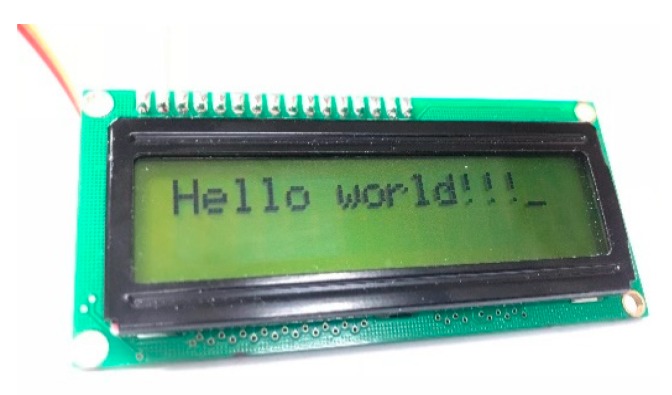
LCD showing the corresponding text.

The in situ tasking application we design is a remotely-controllable LCD and LED device (Figure 14). The device is installed with a Parallax 2 × 16 Serial LCD and a LED. The device periodically sends UpdateStatus requests to update the gateway’s IP address on a service. Please note that the frequency of sending UpdateStatus requests depends on device mobilities, which can be set when constructing devices. For an in situ device, a lower frequency should be enough to make sure the device is accessible via a connected gateway. Algorithm A2 shows the description file of this device. Algorism 5 and 6 show example tasks from the service to turn on the LED and display text on the LCD monitor, respectively. Figure 15 and Figure 16 are results triggered by the tasks.

**Algorithm 5.** An example task to turn on the LED.

{
   "TaskingCapability": {
     "@iot.id": 33 
   }, 
   "input": [ 
     { 
        "ParameterID": "on",
        "Value": *true* 
     } 
   ]
}


**Algorithm 6.** An example task to display text on the LCD monitor.

{ 
   "TaskingCapability": {
     "@iot.id": 34
   }, 
   "input": [
     { 
       "ParameterID": "msg", 
       "Value": "*Hello world!!!*"
     }
   ]
 }


### 4.3. Mobile Sensing Application

**Figure 17:**
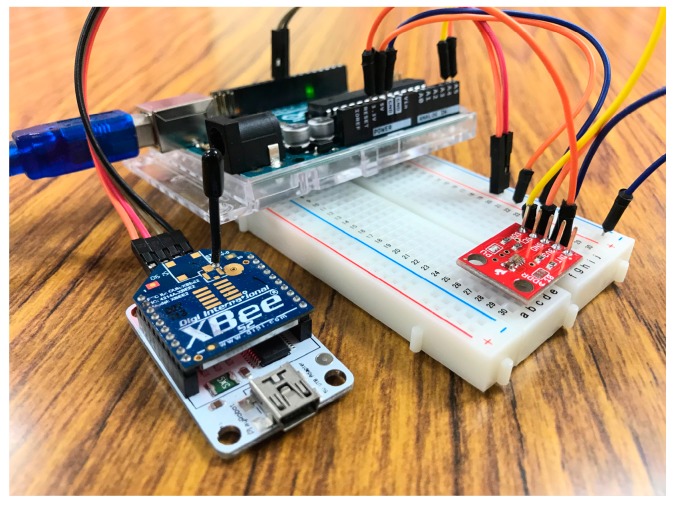
The mobile luminosity-monitoring device.

**Figure 18:**
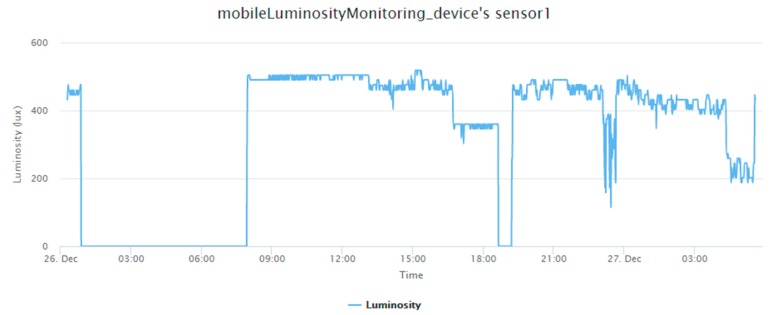
The luminosity observations of the mobile luminosity-monitoring device.

The mobile sensing application we designed is a mobile luminosity-monitoring device (Figure 17), which is installed with a TSL2561 luminosity sensor (TAOS Inc., Plano, TX, USA). The device periodically captures luminosity observations and sends observations through UploadObs requests. When this mobile device connects to a new gateway, the registration procedure will be triggered by UploadObs requests, where the getaway will perform the registration process and upload observations. Algorithm A3 is the description file of this device. Figure 18 shows the observations retrieved from the SensorThings API service.

### 4.4. Mobile Tasking Application

**Figure 19:**
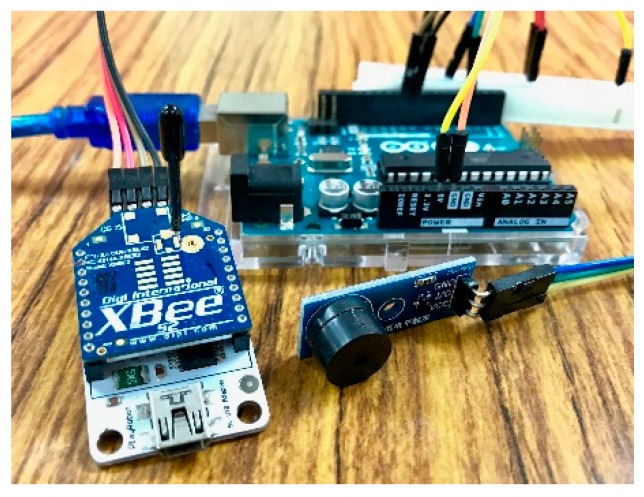
The remotely-controllable buzzer device.

The mobile tasking application we designed is a remotely-controllable buzzer. The device is installed with an MH-FMD passive buzzer module (Figure 19). The device periodically sends UpdateStatus requests to trigger the update of a connected gateway’s IP address to the SensorThings API service. As mentioned earlier, the frequency of UpdateStatus requests could set higher for mobile devices in order to make sure that the SensorThings API service knows the currently-connected gateway. Algorithm A4 shows the description file of this device. Algorithm 7 shows an example task from the service to turn on the buzzer.

**Algorithm 7.** An example task to ring the buzzer five times.

{
   "TaskingCapability":{
      "@iot.id":41
   },
   "input":[
      {
          "ParameterID": "buzz_time", 
          "Value": 5 
      }
    ]
 }


### 4.5. A Real-world Experiment—Underground Metro Mall Environment Monitoring

Besides the aforementioned applications demonstrating the capabilities of the proposed solution, we have also conducted a real-world experiment to evaluate the performance of the proposed solution. This experiment is an indoor monitoring application, where the testing site is at the East Metro Mall in Taipei. The East Metro Mall is a 725 m long underground public space with many restaurants and stores, which is usually crowded during rush hours, meal times, and weekends.

**Figure 20:**
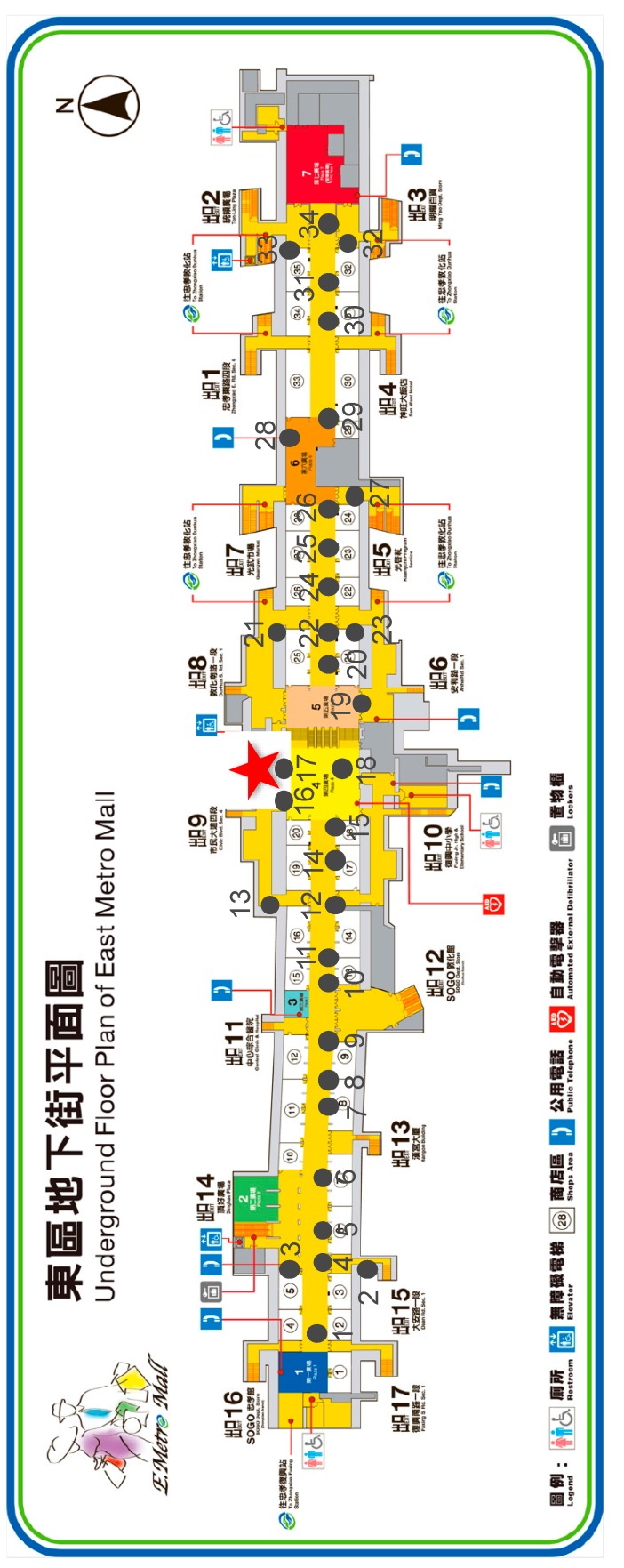
The distribution of the sensors and the gateway of the real-world experiment.

**Figure 21:**
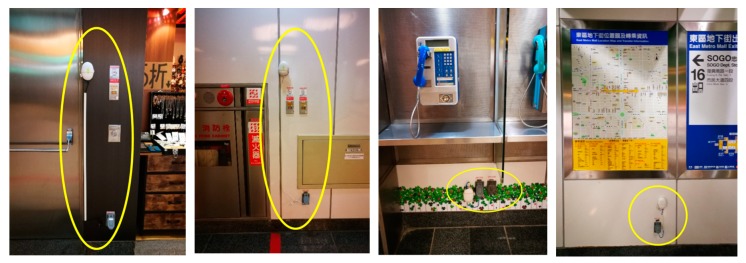
Photos of the deployed devices.

We applied the Arduino Uno, DHT22 temperature and relative humidity sensor, and XBee communication module to construct 34 SW-IoT devices. With the message-forwarding capability of ZigBee, we only deployed one gateway (i.e., a laptop) to receive sensor observations. Figure 20 shows the floor plan and the locations of sensors (i.e., black dots) and the gateway (i.e., red star). The number besides each black dot represents the ID of each device. Figure 21 shows some photos of the deployed devices, where the white boxes contain the devices.

The time period of the testing is from 27 September 2018 to 2 November 2018, where the sampling frequency for each device was set as 15 min. According to the sampling rate, we should receive 117,504 observations in the 37 days of the experiment. However, the gateway was accidently turned off on 11 October 2018. By excluding that day, we should receive 117,540 observations for both temperature and relative humidity. In reality, we have received 108,328 temperature observations and 108,328 relative humidity observations. Therefore, the overall successful return rate is 92.19%.

**Figure 22:**
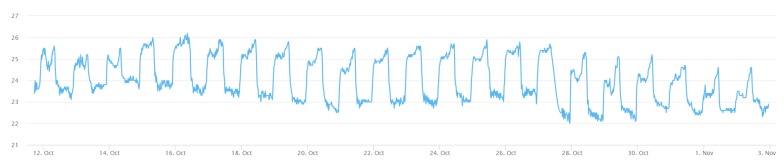
The temperature observations from the device 24 between 12 October 2018 to 2 November 2018.

**Figure 23:**
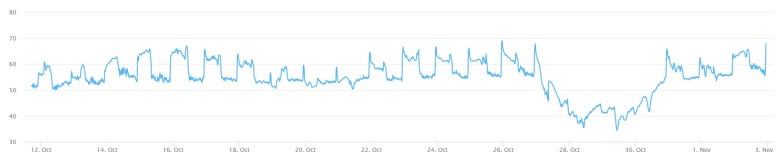
The humidity observations from the device 24 between 12 October 2018 to 2 November 2018.

All the devices can successfully upload sensor observations periodically. Figure 22 and Figure 23 show the time-series observations of device 24 between 12 October 2018 to 2 November 2018 retrieved from the SensorThings API service. As the temperature is ranging between 22 to 26 degree Celsius, it shows a periodical trend that fits with the schedule of air conditioning. On the other hand, while the relative humidity shows a similar behavior with the temperature, it also captured the dry days from 10 October 2018 to 30 October 2018.

## 5. Conclusions and Future Work

This research proposes the IoT-PNP solution that extends the SensorThings API ecosystem to the gateway layer and the device layer to construct a unified SW-IoT architecture. To be specific, we first define a description file for devices to describe its metadata and capabilities. And then with the designed gateway and device operations for wireless communication protocols, such as ZigBee in this research, devices can automatically discover and connect to local gateways. Finally, the proposed automatic registration procedures for sensing and tasking capabilities that can satisfy different scenarios, including new devices, new gateways, and mobile devices.

In order to demonstrate the feasibility of the proposed solution, we have implemented four example applications considering in situ and mobile devices, and their sensing and tasking capabilities. As shown in the experimental results, as long as the embedded devices support the proposed IoT-PNP solution, all the designed applications can be achieved without any human intervention. Overall, we believe that this research provides a valid solution to construct a unified SW-IoT infrastructure and could consequently achieve the SW-IoT vision.

In terms of future work, as SW-IoT embedded devices could support different local wireless communication protocols, such as Bluetooth, LoRaWAN, NB-IoT, etc. we believe that the proposed idea can still be followed with consideration of the characteristics of different protocols. For example, data transmission rates of LoRaWAN, NB-IoT, and Sigfox may not be enough to support the transmission of description files presented in this paper. Possible solutions are to separate a description file into several pieces or to store a description file on the cloud and instruct gateways to retrieve the description file from the cloud. How to apply the IoT-PNP concept to different wireless communication protocols would be an interesting research topic. Finally, we will seek opportunities to apply the proposed IoT-PNP in real-world SW-IoT systems to further demonstrate this solution.

## Figures and Tables

**Table 1:** IoT-Plug and Play (PNP) ZigBee network configurations.

Parameter	Configuration
extended PAN ID (64-bit)	14,647
destination extended address (64-bit)	Coordinator: 0xFFFF
	Router/End device: 0

**Table 2:** Operation properties.

Property	Description
operation	The operation name that a gateway or a device tries to invoke
device_ID	The unique identifier (UID) of the device
service_URL	The service URL to register device resources
msg_body	The corresponding content of each operation

**Table 3:** UploadObs operation.

Operation	UploadObs
functionality	1. Initiate registration procedure of sensing capability 2. Receive sensor observations or new locations from a device
request format	{ "operation": "UploadObs", "device_ID": "<device_ID>", "msg_body": [ { "name": "<datastream_1>", "observation": <obs1> }, { "name": "<datastream_2>", "observation": <obs2> }, { "lat": "<latitude>", "lon": <longitude> } ] }

**Table 4:** UpdateStatus operation.

Operation	UpdateStatus
functionality	1. Initiate registration procedure of tasking capability 2. Update the gateway URL to the service
request format	{ "operation": "UpdateStatus", "device_ID": "<device_ID>" }

**Table 5:** SendServURL operation.

Operation	SendServURL
functionality	Receive the service URL from a device
request format	{ "operation": "SendServURL", "device_ID": "<device_ID>", "service_URL": "<service_URL>" }

**Table 6:** SendDesc operation.

Operation	SendDesc
functionality	Receive the description file from a device
request format	{ "operation": "SendDesc", "device_ID": "<device_ID>", "msg_body ": "<description_file>" }

**Table 7:** SendServURLandDesc operation.

Operation	SendServURLandDesc
functionality	Receive the service URL and the description file from a device
request format	{ "operation": "SendServURLandDesc", "device_ID": "<device_ID>", "service_URL": "<service_URL>", "msg_body ": "<description_file>" }

**Table 8:** GetServURL operation.

Operation	GetServURL
functionality	A gateway asks for the service URL that this device should be registered to
request format	{ "operation": "GetServURL", "device_ID": "<device_ID>" }

**Table 9:** GetDesc operation.

Operation	GetDesc
functionality	A gateway asks for the description file of this device
request format	{ "operation": "GetDesc", "device_ID": "<device_ID>" }

**Table 10:** GetServURLandDesc operation.

Operation	GetServURLandDesc
functionality	A gateway asks for the service URL and the description file
request format	{ "operation": "GetServURLandDesc", "device_ID": "<device_ID>" }

**Table 11:** Confirm operation.

Operation	Confirm
functionality	A gateway acknowledges that the registration procedure is finished
request format	{ "operation": "Confirm", "device_ID": "<device_ID>" }

## Appendix A

**Algorithm A1.** The description file of the indoor monitoring device.
{ "name": "IndoorMonitoring_device", "description": "CSRSR’s sensor", "properties": { "UID": "MY_DEVICE00001" }, "Locations": [ { "name": "CSRSR", "description": "CSRSR", "encodingType": "application/vnd.geo+json", "location": { "type": "Point", "coordinates": [ 24.967765, 121.187035 ] } } ], "Datastreams": [ { "name": "sensor1", "description": "Temperature", "observationType": "http://www.opengis.net/def/observationType/OGC-OM/2.0/OM_Measurement", "unitOfMeasurement": { "name": "degree Celsius", "symbol": "degree Celsius", "definition": "http://unitsofmeasure.org/ucum.html#para-30" }, "Sensor": { "name": "tempSensor", "description": "Thermometer", "encodingType": "text/html", "metadata": "DHT22" }, "ObservedProperty": { "name": "air_temperature", "description": "Temperature of air in situ.", "definition": "http://mmisw.org/ont/ioos/parameter/air_temperature" } }, { "name": "sensor2", "description": "Humidity", "observationType": "http://www.opengis.net/def/observationType/OGC-OM/2.0/OM_Measurement", "unitOfMeasurement": { "name": "Percentage", "symbol": "%", "definition": "http://unitsofmeasure.org/ucum.html#para-29" }, "Sensor": { "name": "humiSensor", "description": "Hygrometer", "encodingType": "text/html", "metadata": "DHT22" }, "ObservedProperty": { "name": "humidity", "description": "Humidity of air in situ.", "definition": "http://mmisw.org/ont/ioos/parameter/relative_humidity" } } ] }

**Algorithm A2.** The description file of the remotely-controllable LCD and LED device.
{ "name": "remotelyControlled_LCD_device", "description": "CSRSR’s sensor", "properties": { "UID": "MY_DEVICE00002" }, "Locations": [ { "name": "CSRSR", "description": "CSRSR", "encodingType": "application/vnd.geo+json", "location": { "type": "Point", "coordinates": [ 24.967765, 121.187035 ] } } ], "TaskingCapabilities": [ { "name": "LightBulb", "description": "", "parameters": [ { "parameterID": "on", "description": "turn on or off", "use": "Mandatory", "definition": { "inputType": "Boolean", "unitOfMeasurement": "Status", "allowedValues": [ { "value": true, "description": "turn on" }, { "value": false, "description": "turn off" } ] } } ], "zigbeeProtocol": { "extendedAddressHigh": "0013A200", "extendedAddressLow": "40BF8550", "messageDataType": "application/text", "messageBody": "MY_DEVICE00002:LightBulb:{on}" }, "Actuator": { "name": "LED", "description": "a controllable LED.", "encodingType": "application/text", "metadata": "LED" } }, { "name": "LCD", "description": "A LCD monitor that can display text messages.", "parameters": [ { "parameterID": "msg", "description": "text", "use": "Mandatory", "definition": { "inputType": "String", "unitOfMeasurement": "Status", "allowedValues": [ { "description": "String" } ] } } ], "zigbeeProtocol": { "extendedAddressHigh": "0013A200", "extendedAddressLow": "40BF8550", "messageDataType": "application/text", "messageBody": "MY_DEVICE00002:LCD:{msg}" }, "Actuator": { "name": "Paralax LCD monitor", "description": "a controllable LCD monitor which can print text.", "encodingType": "application/text", "metadata": "Parallax 2*16 Serial LCD" } } ] }

**Algorithm A3.** The description file of the mobile luminosity-monitoring device.
{ "name": "mobileLuminosityMonitoring_device", "description": "CSRSR’s sensor", "properties": { "UID": "MY_DEVICE00003" }, "Locations": [ { "name": "CSRSR", "description": "CSRSR", "encodingType": "application/vnd.geo+json", "location": { "type": "Point", "coordinates": [ 24.967757, 121.187112 ] } } ], "Datastreams": [ { "name": "sensor1", "description": "Luminosity", "observationType": "http://www.opengis.net/def/observationType/OGC-OM/2.0/OM_Measurement", "unitOfMeasurement": { "name": "Lux", "symbol": "lux", "definition": "http://unitsofmeasure.org/ucum.html#para-30" }, "Sensor": { "name": "TSL2561", "description": "Luminosity_Sensor", "encodingType": "text/html", "metadata": "TSL2561" }, "ObservedProperty": { "name": "Luminosity", "definition": "https://ci.mines-stetienne.fr/seas/ZoneLightingOntology-1.0#luminosity", "description": "Luminosity." } } ] }

**Algorithm A4.** The description file of the remotely-controllable buzzer device.
{ "name": "remotelyControlledBuzzer_device", "description": "CSRSR’s sensor", "properties": { "UID": "MY_DEVICE00004" }, "Locations": [ { "name": "CSRSR", "description": "CSRSR", "encodingType": "application/vnd.geo+json", "location": { "type": "Point", "coordinates": [ 24.967757, 121.187112 ] } } ], "TaskingCapabilities": [ { "name": "buzzer", "description": "", "parameters": [ { "parameterID": "buzz_time", "description": "ring buzzer several times", "use": "Mandatory", "definition": { "inputType": "Integer", "unitOfMeasurement": "times", "allowedValues": [ { "Max": 10, "Min": 1 } ] } } ], "zigbeeProtocol": { "addressingSH": "0013A200", "addressingSL": "40BF8550", "messageDataType": "application/text", "messageBody": "MY_DEVICE00024:buzzer:{buzz_time}" }, "Actuator": { "name": "MH-FMD buzzer module", "description": "a controllable buzzer.", "encodingType": "application/text", "metadata": "MH-FMD" } } ] }
